# AAV-Vectored Expression of the Vascular Normalizing Agents 3TSR and Fc3TSR, and the Anti-Angiogenic Bevacizumab Extends Survival in a Murine Model of End-Stage Epithelial Ovarian Carcinoma

**DOI:** 10.3390/biomedicines10020362

**Published:** 2022-02-02

**Authors:** Ashley A. Stegelmeier, Lisa A. Santry, Matthew M. Guilleman, Kathy Matuszewska, Jessica A. Minott, Jacob G. E. Yates, Brenna A. Y. Stevens, Sylvia P. Thomas, Sierra Vanderkamp, Kiersten Hanada, Yanlong Pei, Amira D. Rghei, Jacob P. van Vloten, Madison Pereira, Brad Thompson, Pierre P. Major, James J. Petrik, Byram W. Bridle, Sarah K. Wootton

**Affiliations:** 1 Department of Pathobiology, University of Guelph, Guelph, ON N1G 2W1, Canada; astegelm@uoguelph.ca (A.A.S.); lsantry@elevate.bio (L.A.S.); mguilleman@elevate.bio (M.M.G.); minott@uoguelph.ca (J.A.M.); jyates01@uoguelph.ca (J.G.E.Y.); bsteve04@uoguelph.ca (B.A.Y.S.); sthoma13@uoguelph.ca (S.P.T.); vanderka@uoguelph.ca (S.V.); khanada@uoguelph.ca (K.H.); ypei@ovc.uoguelph.ca (Y.P.); arghei@uoguelph.ca (A.D.R.); vanVloten.Jacob@mayo.edu (J.P.v.V.); bridle@uoguelph.ca (B.W.B.); 2 Department of Biomedical Sciences, University of Guelph, Guelph, ON N1G 2W1, Canada; kmatusze@uoguelph.ca (K.M.); mperei02@uoguelph.ca (M.P.); jpetrik@uoguelph.ca (J.J.P.); 3 Avamab Pharma Inc., Calgary, AB T3E 6L1, Canada; bt@kickshawventures.com; 4 Juravinski Cancer Centre, 699 Concession Street, Hamilton, ON L8V 5C2, Canada; majorp@hhsc.ca

**Keywords:** ovarian cancer, AOaV-1, gene therapy, oncolytic virotherapy, adeno-associated virus (AAV), vascular normalization

## Abstract

Epithelial ovarian cancer is the deadliest gynecological malignancy. The lack of effective treatments highlights the need for novel therapeutic interventions. The aim of this study was to investigate whether sustained adeno-associated virus (AAV) vector-mediated expression of vascular normalizing agents 3TSR and Fc3TSR and the antiangiogenic monoclonal antibody, Bevacizumab, with or without oncolytic virus treatment would improve survival in an orthotopic syngeneic mouse model of epithelial ovarian carcinoma. AAV vectors were administered 40 days post-tumor implantation and combined with oncolytic avian orthoavulavirus-1 (AOaV-1) 20 days later, at the peak of AAV-transgene expression, to ascertain whether survival could be extended. Flow cytometry conducted on blood samples, taken at an acute time point post-AOaV-1 administration (36 h), revealed a significant increase in activated NK cells in the blood of all mice that received AOaV-1. T cell analysis revealed a significant increase in CD8^+^ tumor specific T cells in the blood of AAV-Bevacizumab+AOaV-1 treated mice compared to control mice 10 days post AOaV-1 administration. Immunohistochemical staining of primary tumors harvested from a subset of mice euthanized 90 days post tumor implantation, when mice typically have large primary tumors, secondary peritoneal lesions, and extensive ascites fluid production, revealed that AAV-3TSR, AAV-Fc3TSR+AOaV-1, or AAV-Bevacizumab+AOaV-1 treated mice had significantly more tumor-infiltrating CD8^+^ T cells than PBS controls. Despite AAV-mediated transgene expression waning faster in tumor-bearing mice than in non-tumor bearing mice, all three of the AAV therapies significantly extended survival compared to control mice; with AAV-Bevacizumab performing the best in this model. However, combining AAV therapies with a single dose of AOaV-1 did not lead to significant extensions in survival compared to AAV therapies on their own, suggesting that additional doses of AOaV-1 may be required to improve efficacy in this model. These results suggest that vectorizing anti-angiogenic and vascular normalizing agents is a viable therapeutic option that warrants further investigation, including optimizing combination therapies.

## 1. Introduction

Ovarian cancer is the seventh most commonly diagnosed cancer among women in the world [1] and accounts for more deaths than any other cancer of the female reproductive system [2]. Prognosis remains poor due to late stage diagnosis [3] and the fact that the primary tumor readily metastasizes to other organs in the peritoneal cavity [4]. Despite an increased understanding of the pathogenesis of ovarian cancer, mortality rates have not changed significantly over the past four decades. Thus, novel therapeutic strategies are needed to more effectively treat advanced-stage disease.

Rapidly growing tumors create their own blood vessels to supply them with nutrients and oxygen to support their high metabolic needs [5]. Tumor vasculature is known to be highly abnormal due to unbalanced, local overexpression of a small number of growth factors, particularly vascular endothelial growth factor-A (VEGF-A) [6]. Treatment modalities that block VEGF and its pro-angiogenic functions have been validated in various types of human cancers and neoplastic tissues [7]. The an anti-VEGF monoclonal antibody (mAb), Avastin^®^ (bevacizumab), is an approved treatment for multiple types of cancers including lung, colon, glioblastoma, and renal-cell carcinoma [8]. Bevacizumab works by selectively interacting with two binding sites on all circulating isoforms of VEGF-A, thereby inhibiting the binding of VEGF-A to the epithelial cell surface receptors, VEGRF-1 and VEGRF-2 [9]. When VEGF-A cannot bind to its receptors, tumor blood supply and interstitial pressure are reduced, while chemotherapy delivery and vascular permeability are increased [10]. Although Bevacizumab treatment increases progression-free survival in women with ovarian cancer [11,12,13], transient and low peritoneal drug levels can influence treatment efficacy [14]. Additionally, since tumor cells constantly express VEGF [15], a persistent delivery of bevacizumab may be needed to prevent revascularization associated with anti-VEGF therapy withdrawal. Indeed, maintenance Bevacizumab treatment has been demonstrated to have better efficacy with acceptable toxicity in clinical trials [12,16], but this requires repetitive slow intravenous infusion [17]. Using a gene therapy vector to constitutively express Bevacizumab represents one way to mediate sustained expression and reduce the peaks and troughs associated with systemic delivery of recombinant mAbs [18,19].

Vascular normalizing agents, on the other hand, normalize disorganized, tortuous tumor vasculature networks leading to improved tumor perfusion and increased uptake of anti-cancer drugs and immunotherapies to previously inaccessible regions of the tumor [20,21]. Thrombospondin-1 (TSP-1) is a natural inhibitor of neovascularization and tumorigenesis that activates transforming growth factor beta (TGF-β) and inhibits angiogenesis by interacting with CD36 on endothelial cells [22,23,24,25]. The three type I repeats (3TSR) of TSP-1 have been associated with the majority of the anti-angiogenic functions of the protein. 3TSR has potent normalizing effects on tumor vasculature, which in turn promotes delivery of therapeutics and immune cells to tumors [26]. When administered in a mouse model of epithelial ovarian cancer (EOC), 3TSR induces apoptosis, slows primary tumor growth, and reduces the number of secondary metastases [27,28]. However, the half-life of 3TSR is very short [29] thus daily injections are needed to induce an optimal biological effect. Advances that permit longer term expression of 3TSR, including the addition of an Fc domain, serve to increase duration of expression, but still require weekly administrations [30]. Thus, the vectorized expression of 3TSR or Fc3TSR may serve to improve this promising treatment modality [28].

Adeno-associated virus (AAV) gene therapy vectors are safe [31] and effective in vivo gene delivery vehicles that promote long-term transgene expression [32,33], with applications to cancer treatment [34,35,36,37,38,39,40]. AAV has been used to express a wide array of anti-cancer agents including endostatin [41,42], angiostatin [43,44], pigment epithelium-derived factor (PEDF) [45], and VEGF-Trap [46,47], to name a few. Results from these and other studies indicate that systemic expression of antiangiogenic agents from AAV represents a potentially effective cancer treatment [48], either alone or in combination with other therapies such as chemotherapeutic drugs [18,49].

Although numerous AAV serotypes with variable tropism exist [33], the novel triple-mutant AAV6.2FF capsid, which possesses an amino acid substitution (F129L) that facilitates heparin binding (AAV6.2) at the cell surface [50], and two mutations (Y445F, Y731F) that abrogate ubiquitin-mediated degradation [51], represents and ideal candidate for in vivo expression of antiangiogenic compounds due to its ability to mediate rapid and sustained transgene expression following intramuscular administration [52,53]. In a head to head comparison, AAV6.2FF outperformed other commonly used AAV capsids including AAV6, AAV8, AAV9, and DJ, by expressing significantly more luciferase in the first 14 days post-administration [54]. Additionally, a recently published safety and tolerability study in rodents and sheep demonstrates that this rationally engineered AAV6.2FF vector is safe and effective in multiple species.

Given that 3TSR requires daily injections due to its short half-life, and because maintenance Bevacizumab treatment results in better efficacy, we hypothesized that AAV6.2FF-mediated expression of these antiangiogenic compounds would promote sustained levels in the blood without the need for daily or frequent injections and less pharmacokinetic fluctuation. Here, we tested the hypothesis that a single administration of AAV expressing Bevacizumab, 3TSR, or a modified version of 3TSR containing the Fc domain from human IgG (Fc3TSR) [30] will result in sustained serum-expression levels and extended survival in a syngeneic orthotopic mouse model of EOC. Additionally, we investigate whether administration of oncolytic avian orthoavulavirus-1 (AOaV-1) [55,56] in combination with the AAV therapies will serve to further increase survival in this model.

## 2. Materials and Methods

### 2.1. Ethics

All animal experiments were approved by the University of Guelph’s Animal Care Committee and conducted in accordance with Canadian Council on Animal Care (CCAC). Eight-week-old C57BL/6 female mice were purchased from Charles River Laboratories (Saint Constant, QC, Canada) and housed four per cage at the Animal Isolation Facility, University of Guelph. Mice were acclimated for 1 week prior to experimentation.

### 2.2. AAV Vector Construction and Virus Production

**Figure 1:**
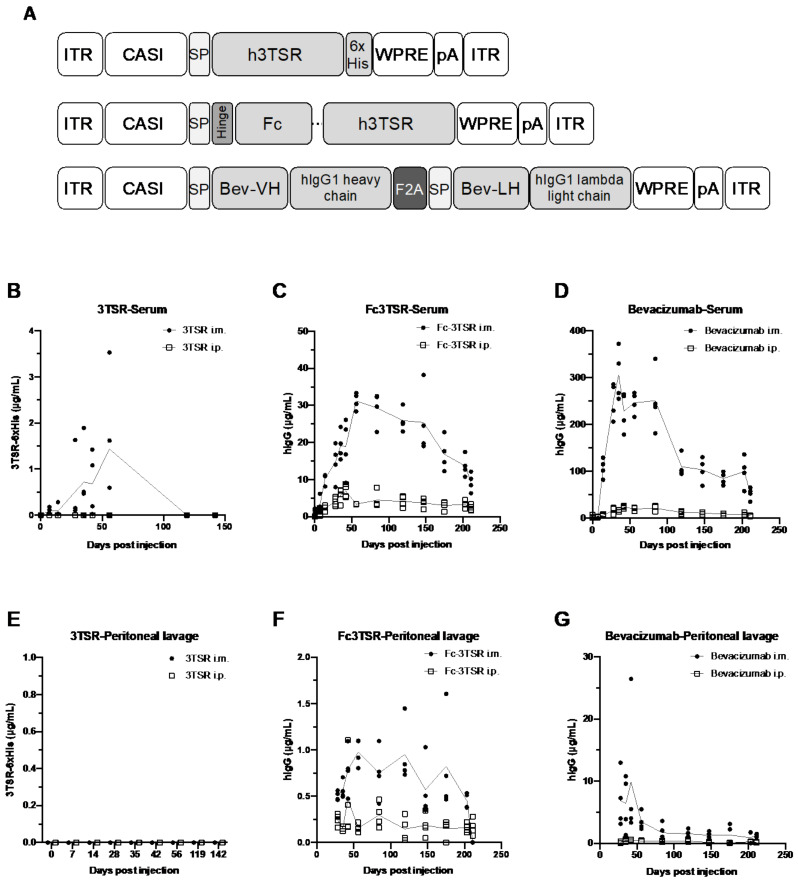
Schematic representation of the AAV genomes and kinetics of therapeutic transgene expression in the serum and peritoneal cavity of non-tumor bearing C57BL/6 mice. (**A**) Schematic of the three AAV genomes engineered to express 3TSR, Fc3TSR, and Bevacizumab. Transgenes were expressed under the control of the ubiquitous CASI promoter (CASI) [57] and contained a human growth hormone signal peptide (SP) to enable protein secretion. A hinge followed by the human IgG1 Fc domain was cloned upstream of human 3TSR to create Fc3TSR. A C-terminal His tag (6xHis) was added to 3TSR to facilitate detection and quantification. The heavy and light chains of Bevacizumab were separated by a furin F2A self-cleaving peptide (F2A). All three vector genomes contain a WPRE followed by a simian virus 40 polyadenylation signal (pA). AAV genomes were flanked by AAV2 inverted terminal repeat (ITR) sequences. Fc, fragment crystallizable; 3TSR, thrombospondin-1 type I repeats; Bev, bevacizumab; VH, variable heavy chain; VL, variable light chain; WPRE, Woodchuck Hepatitis Virus Posttranscriptional Regulatory Element. (**B**–**G**) C57BL/6 mice were administered 1 × 10^11^ vg of AAV-3TSR, AAV-Fc3TSR, or AAV-Bevacizumab i.m. (**A**–**C**) or i.p. (**E**–**G**). Saphenous bleeds and peritoneal washes were conducted over time to monitor transgene expression. A quantitative human IgG ELISA was used to quantify Fc3TSR and Bevacizumab expression, whereas a His-Tag ELISA was used to quantify 3TSR expression.

The heavy (KX119517.1) and light chains of Bevacizumab (KX119516.1), separated by a self-cleaving F2A peptide, were synthesized as a full length human IgG antibody and cloned into the KpnI-XbaI site of pACASI-MCS-WPRE which contains a strong constitutive CASI promoter [57], a WPRE element [58] and an SV40 polyA signal surrounded by AAV2 inverted terminal repeats. 3TSR (amino acids to 378 to 548) from human TSP-1 (NM_003246.4) synthesized to contain a 6xHis tag at the C-terminus was cloned into the KpnI-XbaI site of pACASI-MCS-WPRE. Fc3TSR was synthesized with a human growth hormone (HGH)-signal peptide, hinge, CH2, CH3 domains of hIgG1, and a flexible (Gly4Ser)4 linker upstream of human 3TSR. All-gene synthesis was performed by GenScript (Piscataway, NJ, USA). See Figure 1A for schematic of AAV vector genomes used in this study. AAV genomes were packaged into the AAV6.2FF capsid by co-transfection of human embryonic kidney 293 cells (ATCC CRL-1573), confirmed mycoplasma free using the MycoAlert mycoplasma detection kit (Lonza Cat. #LT07-118; Morrisville, NC) with genome and packaging plasmids as previously described [59]. AAV6.2FF vectors were purified using heparin columns [60] and vector titers were determined by quantitative polymerase chain reaction analysis as previously described [61].

### 2.3. AOaV-1 Production and Purification

The full-length cDNA genome of AOaV-1 was synthesized based on GenBank accession AF077761.1 and containing a GFP reporter gene between the P and M genes. An F3AA polybasic cleavage site [62] and leucine to alanine mutation at position 289 were introduced into the fusion protein [63] by site-directed mutagenesis to generate pAOaV-1 (F3aa)-L289A-GFP. Recombinant AOaV-1-GFP was rescued using Modified Vaccinia Ankara (MVA) expressing T7 RNA polymerase, a kind gift from Dr. Bernard Moss, as amplified in egg and purified as described previously [56]. Mice were administered AOaV-1-GFP intravenously at a dose of 1 × 10^8^ PFU/mouse.

### 2.4. Transgene Expression Monitoring

Eight-week-old female C57BL/6 mice were used to monitor the duration of AAV-mediated transgene expression in the serum and peritoneal cavity after intramuscular (i.m.) or intraperitoneal (i.p.) administration. Four mice were included in each of the following experimental groups: AAV-Fc3TSR i.m. injection route, AAV-Fc3TSR i.p. injection route, AAV-3TSR i.m. injection route, AAV-3TSR i.p. injection route, AAV-Bevacizumab i.m. injection route, and AAV-Bevacizumab i.p. injection route for a total of 24 mice. Mice were administered 1 × 10^11^ vg of AAV, either diluted to a total volume 40 µL PBS for the i.m. injections or 200 µL total volume for the i.p. injections. Saphenous vein bleeds and peritoneal washes were initially conducted once a week, followed by once per month after 56 days. The experiment was ended on day 211, when low levels of Fc3TSR and Bevacizumab expression remained quantifiable.

### 2.5. ID8 Ovarian Epithelial Cancer Model

Spontaneously transformed murine ovarian surface epithelial cells (ID8 cells), generously provided by Drs. K. Roby and P. Terranova, Kansas State University, Manhattan, KS, USA) were cultured in DMEM supplemented with 10% fetal bovine serum (FBS), 2% L-glutamine and 1% penicillin-streptomycin. 1 × 10^6^ ID8 cells suspended in 6 µL PBS were injected directly into the left ovarian bursa of 112 mice as described previously [64]. At 50 days post ID8 tumor cell implantation, groups of mice received AAV-3TSR (1 × 10^11^ vg/mouse), AAV-Fc3TSR (1 × 10^11^ vg/mouse), AAV-Bevacizumab (1 × 10^10^ vg/mouse to correct for magnitude higher transgene expression levels) or PBS via i.m administration. Fourteen days later, half the mice in each group was administered 1 × 10^8^ PFU AOaV-1 i.v. Of the 112 mice allotted for this study, one died shortly after arrival, one in the AAV-Bevacizumab survival group died post operation and another in the Fc3TSR+AOaV-1 group was crushed by her cage mates. These mice were therefore removed from the study prior to receiving any treatment, leaving 62 mice in the survival study and 47 mice in the 90-day necropsy study.

Throughout the following months, mice were monitored by the animal care staff daily, with additional monitoring conducted by the researchers biweekly, including weekly recording of weight. Monitoring increased when the first signs of ascites appeared in mice (~day 75 in the earliest mice), characterized by expanding torso size. Mice with ascites were drained ~1 time per week as needed using a 26-gauge needle, and generally reached endpoint after ~5 drains. The ascites were quantified and centrifuged at 5500 rpm for 5 min. The cell-free supernatant was separated and frozen at −20 °C for future analysis. At day 90 post tumor cell implantation, 6 mice from each group were euthanized to quantify disease progression. Note that three mice died prior to the day 90 endpoint and were not included in the final analysis. From each mouse the ascites fluid was harvested and measured, the primary tumor was weighed, and the secondary tumors in the peritoneal cavity enumerated. The primary tumor and tumor draining lymph node were fixed in 10% formalin overnight. The next day, tissue samples were rinsed three times in PBS and stored in 70% ethanol. Fixed samples were embedded in paraffin wax, and sectioned onto slides. Each tissue was sectioned with a minimum of two sections per slide, seven slides per tissue. Slides were stained using the Hydroxyprobe kit according to the manufacturer’s protocol (HP1-200; Hydroxyprobe Inc., Burlington, MA, USA) to assess hypoxia in the tumors and lymph nodes. Immunohistochemistry was conducted to ascertain CD8^+^ T cell and B cell infiltration as described previously [65]. Mice in the survival study were monitored daily, increasing to four times per day as disease progressed. Endpoint was defined as a combination of consistent weight loss, reduction in movement, lethargy, hunched posture, and ruffled fur. Mice at endpoint were humanely euthanized using isoflurane and cervical dislocation and necropsies were immediately conducted to characterize disease parameters.

### 2.6. Flow Cytometry

Blood was collected for flow cytometry via non-lethal retro-orbital bleeds. One set of samples was taken 36 h after AOaV-1 administration for NK analysis, while a second blood draw occurred 10 days after AOaV-1 administration to quantify tumor specific T cells as described [66,67]. All centrifugation steps were performed at 500× *g* for 5 min at 4 °C. Blood was mixed with ACK lysis buffer (Per 1 L: 8.29 g NH_4_Cl, 1 g KHCO_3_, 37.2 mg Na_2_EDTA, H_2_O to 1 L total volume, pH 7.2–7.4) and incubated for 5 min at room temperature to lyse red blood cells. The mixture was diluted in Hank’s Balanced Buffered Salt (HBSS; HyClone; Cat. #SH30588.02) solution, centrifuged, and the process was repeated one more time. Remaining white blood cells were incubated with Fc block (BioLegend, San Diego, CA, USA; Clone 93; Cat. #101320) for 15 min at 4 °C. Cells were then rinsed in phosphate-buffered solution (PBS; HyClone; Cat. #SH30256.02) and centrifuged at 500× *g* for 5 min at 4 °C. Surface staining involved diluting antibodies in FACS buffer (500 mL PBS, 2.5 g Bovine serum albumin-HyClone; Cat. #SH40015.01) in a total of 50 µL/well and incubating for 20 min at 4 °C. NK cell panel #1 was composed of anti-CD69 FITC (BD Biosciences, Franklin Lakes, NJ, USA; Clone H1.2F3; Cat. #553236), anti-PD-L1 PE (BD Biosciences; Clone MIH5; Cat. #558091), anti-PD-1 PerCp (BioLegend; Clone RMP1-30; Cat. #109120), anti-CD4 PE-Cy7 (BioLegend; Clone RM4-5; Cat. #100528), anti-NK1.1 APC (BioLegend; Clone PK136; Cat. #108710), anti-CD3 BV421 (BioLegend; Clone 145-2C11; Cat. #100336), anti-CD8 BV510 (BioLegend; Clone 53-6.7; Cat. #100752; 0.125 µL each). NK cell panel #2 was composed of anti-NKp46 PE-Cy7 (BioLegend; Clone 29A1.4; Cat. #137618), anti-NK1.1 APC (BioLegend; Clone PK136; Cat. #108710), anti-NKG2D BV421 (BD Biosciences; Clone CX5; Cat. #562800), anti-CD3 BV510 (BioLegend; Clone 17A2; Cat. #100233). After the incubation, wells were rinsed with PBS twice, and centrifuged after each rinse. Zombie NIR fixable viability dye (BioLegend; Cat. #423106) was diluted 1:1000 and 100 µL/well added. Cells were incubated at 4 °C for 30 min. Stained cells were rinsed twice with PBS and centrifuged. At this stage panel #1 was complete; however, panel #2 required intracellular staining. These samples were fixed in an IC fixation buffer (BioLegend; Cat. #420801) for 20 min at 4 °C, then rinsed in permeabilization buffer (BioLegend; Cat. #421002) twice. An intracellular master mix consisting of anti-Granzyme B-FITC (BioLegend; Clone GB11; Cat. #515403), anti-IFN-γ PE (BioLegend; Clone XMG1.2; Cat. #505808) and permeabilization buffer to a total volume of 25 µL was added to each well and incubated for 20 min at 4 °C. Cells were rinsed twice in permeabilization buffer and centrifuged for 5 min at 4 °C after each rinse. All cells were resuspended in FACS buffer before being run on the BD FACS Canto and visualized using FACS Diva software.

The T cell analysis on blood samples collected 10 days post AOaV-1 injection followed similar steps to the NK staining with the following deviations. The surface-staining panel was comprised of anti-CD69 FITC (BD Biosciences; Clone H1.2F3; Cat. #553236), anti-CD4 PE-Cy7 (BioLegend; Clone RM4-5; Cat. #100528), anti-CD3 BV421 (BioLegend; Clone 145-2C11; Cat. #100336), and anti-CD8 BV510 (BioLegend; Clone 53-6.7; Cat. #100752). The intracellular panel included anti-TNF-α PE (BD Biosciences; Cat. #554419), anti-IFN-γ APC (BioLegend; Clone XMG1.2; Cat. #505808). Samples were split into two wells. To determine the number of tumor specific T cells, one set of samples was added to a U bottom plate seeded with 5 × 10^4^ ID8 cells/well 48 h prior and treated with 100 units murine IFN-γ (eBioscience, SD, USA, Cat. #14-8311-63) per well in 50 µL complete media. Tumor specific T cells were quantified by subtracting the number of TNF-α^+^IFN-γ^+^ T cells from cells not plated in the presence of ID8 cells, as described previously [66].

### 2.7. Tumor-Directed Antibody Responses

Tumor-directed antibody responses were quantified as previously described [66]. Tumor-directed antibody data were analyzed by first subtracting background fluorescent of control wells from each sample. Then, a curve was constructed for each sample using the dilution series. The area under the curve was then calculated for each sample and graphed alongside tumor-bearing but untreated animal controls.

### 2.8. ELISA

Commercial enzyme linked immunoassays (ELISAs) were used to quantify transgene expression levels in mice administered AAV vectors. The Abcam Human IgG ELISA kit (ab195215; Branford, CT, USA) was used to quantify Fc3TSR and Bevacizumab in the plasma and peritoneal washes. The Cell Biolabs His-Tag Protein ELISA (AKR-130; San Diego, CA, USA) was used to quantify 3TSR-6xHis protein concentrations in plasma and peritoneal washes. Lavages were collected by administering 3 mL of sterile PBS into the peritoneal cavity, gently palpating the torso to evenly distribute the liquid, and subsequently removing 1 mL of lavage fluid. Serum was collected via saphenous bleeds, followed by a centrifugation at 5500 rpm for 5 min to obtain serum from the blood sample. All steps were followed according to the manufacturers’ instructions.

### 2.9. Statistical Analyses

Flow cytometry analyses were conducted using FlowJo v. 10. This software was used to define gating (Figure S2) and to export excel files with the proportion and numbers of each cell subset. GraphPad Prism v. 9 was used to analyze all data and generate graphs. Two way ANOVAs were conducted using the multiple comparisons function to calculate significance between experimental groups in the flow cytometry experiments. The Log-rank Mantel-Cox test was used to determine which treatments led to a significant extension in survival. * *p* < 0.05; ** *p* < 0.01; *** *p* < 0.001; **** *p* < 0.0001; ns = not significant.

## 3. Results

### 3.1. AAV-Mediated Expression of 3TSR, Fc3TSR and Bevacizumab Results in Sustained Transgene Expression in the Blood and Peritoneal Cavity, with Intramuscular Administration Resulting in Higher Expression Levels

Groups of eight-week old female C57BL/6 (n = 4) were administered 1 × 10^11^ vg of AAV-3TSR, AAV-Fc3TSR, or AAV-Bevacizumab either intramuscularly (i.m.) or intraperitoneally (i.p.) and transgene expression was monitored for over 200 days. Saphenous bleeds and non-terminal i.p. lavages were conducted on days 0, 7, 14, 28, 35, 42, and 56, after which time the collections were extended to once per month. All three therapeutic transgenes were expressed at higher levels in the serum (Figure 1B–D) following i.m. injection compared to i.p. injection. The concentration of 3TSR in the serum and the peritoneal cavity was markedly lower than Fc3TSR or Bevacizumab (Figure 1B,E) and was only detectable in the serum of mice that received AAV-3TSR i.m., where it peaked on day 56 at 1.4 ± 1.5 μg/mL and was below the level of detection by day 142 (Figure 1B). AAV-Fc3TSR administration displayed intermediate levels of Fc3TSR expression compared to the other therapeutic transgenes (Figure 1C,F). Expression levels peaked on day 56 at 31.2 ± 2.2 μg/mL. Expression was also observed in the serum when the vector was administered i.p.; however, hIgG levels were significantly lower than in the serum. Furthermore, Fc3TSR expression levels in peritoneal lavages of i.m. injected mice peaked at day 56, and were below the level of detection on day 211. The concentration of Fc3TSR in peritoneal lavages of i.p. injected mice peaked on day 42, mimicking the trends observed in the serum samples. Bevacizumab expression from AAV was an order of magnitude higher compared to the other two therapeutic transgenes (Figure 1D,G). When administered i.m., Bevacizumab expression levels in the serum peaked at 306.5 ± 55.2 hIgG μg/mL on day 35, and slowly decreased to 52.8 ± 12.9 IgG μg/mL by day 211. When administered i.p., lower serum levels were quantified compared to i.m. Peritoneal lavages for both injection routes demonstrated similar trends. Based on these results, we administered AAV-Bevacizumab at dose of 1 × 10^10^ vg in the ID8 ovarian cancer experiments so that serum expression levels were of similar magnitude to the other gene therapies.

### 3.2. ID8 Tumor-Bearing Mice Expressing Vectorized Fc3TSR and Treated with Oncolytic AOaV-1 Had Significantly Increased Numbers of Activated NK Cells 36 h Post Treatment

**Figure 2:**
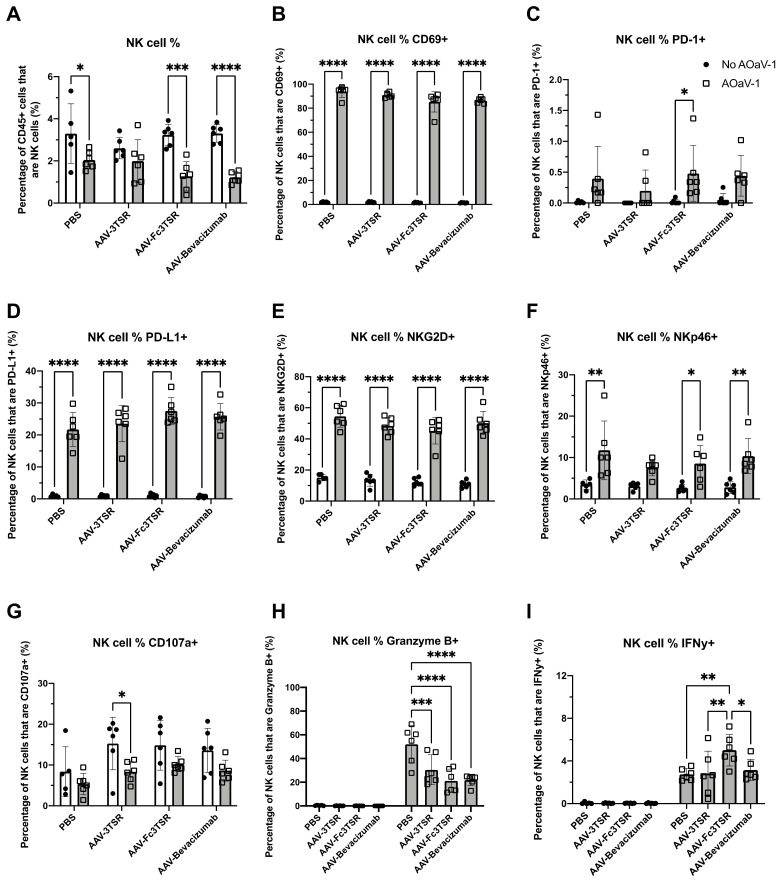
NK cell analysis of blood samples taken 36 h post AOaV-1 administration to mice previously i.m. administered AAV-3TSR (1 × 10^11^ vg), Fc3TSR (1 × 10^11^ vg) or AAV-Bevacizumab (1 × 10^10^ vg). Non-terminal retro-orbital blood samples were obtained 36 h post i.v. administration of 1 × 10^8^ PFU AOaV-1. Red blood cells were lysed, and the remaining cells were stained and analyzed via flow cytometry. (**A**) Percentage of CD45+ cells that are NK cells. Percentage of NK cells expressing (**B**) the early activation marker CD69, (**C**) the immune checkpoint protein programmed cell death protein 1 (PD-1), (**D**) the immune checkpoint protein programmed death ligand 1 (PD-L1), (**E**) the cytotoxic activating receptor NKG2D, (**F**) the natural cytotoxicity receptor NKp46, (**G**) the NK functional marker CD107a, (**H**) the serine protease Granzyme (**B**,**I**) IFN-γ. AOaV-1, avian orthoavulavirus-1; AAV, Adeno-associated virus; PBS, phosphate-buffered saline mock control group; 3TSR, thrombospondin-1 type I repeats; * *p* < 0.05; ** *p* < 0.01; *** *p* < 0.001; **** *p* < 0.0001; ns = not significant. Error bars denote standard deviation.

The ID8 orthotopic mouse model of EOC was employed to evaluate whether AAV-mediated expression of antiangiogenic compounds 3TSR, Fc3TSR, and Bevacizumab either alone or in combination with oncolytic AOaV-1 would improve efficacy in this model of late stage ovarian cancer. A combination of AAV-3TSR (1 × 10^11^ vg/mouse), AAV-Fc3TSR (1 × 10^11^ vg/mouse), and AAV-Bevacizumab (1 × 10^10^ vg/mouse to correct for magnitude higher transgene expression levels), were administered i.m. 40 days post ID8 tumor cell implantation. Twenty days post AAV injection AOaV-1 was administered i.v. (1 × 10^8^ PFU/mouse) to half of the mice. Retro-orbital blood samples were taken 36 h post AOaV-1 administration, at the peak of the NK cell response, to investigate whether the magnitude of the NK cell response in the blood was different in mice expressing 3TSR, Fc3TSR or Bevacizumab. Mice administered AOaV-1 showed evidence of OV-induced leukopenia (Figure 2A) as has been observed previously [67]. There was a significant reduction in the proportion of NK cells in the blood when AOaV-1 was administered for all treatments (*p* < 0.0001–0.0265) except for AAV-3TSR (*p* = 0.4819).

Administration of AOaV-1 significantly increased the expression of multiple surface receptors on NK cells. At 36 h post-AOaV-1 administration, the early activation marker CD69 was significantly upregulated on NK cells (*p* < 0.0001; Figure 2B). PD-1 expression had a far less prominent shift in AOaV-1 treated mice, with significant upregulation only in the AAV-Fc3TSR group (*p* = 0.0481) (Figure 2C). In contrast, there was significant upregulation of PD-L1 on NK cells for all treatments (*p* < 0.0001; Figure 2D). Likewise, the cytotoxic activating receptor NKG2D was upregulated in combination therapies (*p* < 0.0001) (Figure 2E). The natural cytotoxicity receptor NKp46 was also significantly upregulated (*p* = 0.0240–0.0015) in all combination therapies except for AAV-3TSR (*p* = 0.1206) (Figure 2F). The NK functional marker CD107a decreased in monotherapies; however, this change was only significant in the AAV-3TSR combination therapy (*p* = 0.0473) (Figure 2G). This marker has been correlated with the ability of NK cells to lyse target cells and secrete cytokines [68].

The intracellular concentration of Granzyme B and IFN-γ in NK cells varied between mono- and combination therapies, as well as between the combination therapies (Figure 2H,I). Granzyme B was significantly upregulated in all AOaV-1 treated groups (*p* < 0.0001–0.003); however, there were no significant differences between the combination therapies (Figure 2H). IFN-γ was also significantly upregulated after AOaV-1 administration (*p* < 0.001–0.003) (Figure 2I). Within the combination therapies, AAV-Fc3TSR had a significantly higher proportion of IFN-γ^+^ NK cells than either AAV-3TSR (*p* = 0.0029) or AAV-Bevacizumab (*p* = 0.0137).

### 3.3. Tumor Specific TNF-α^+^IFN-γ^+^CD8^+^ T Cells Were Significantly Increased in the AAV-Bevacizumab+AOaV-1 Treatment Group

**Figure 3:**
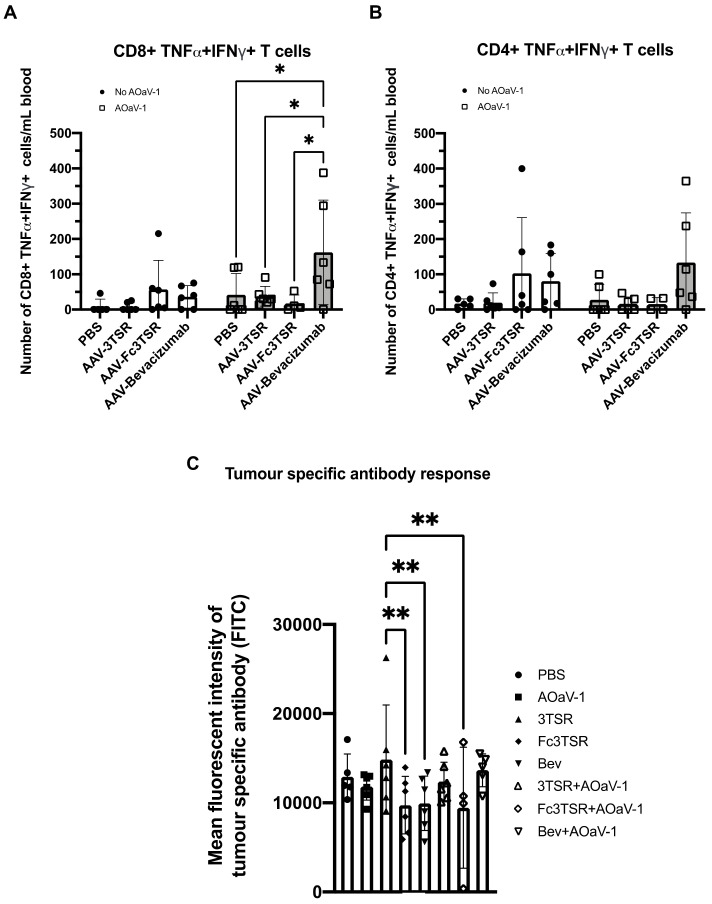
Tumor specific T cell and antibody analysis 10 and 30 days post AOaV-1 administration, respectively. Retro-orbital blood samples were taken 10 days after mice received an i.v. injection of 1 × 10^8^ PFU AOaV-1 or PBS. Red blood cells were lysed, and the remaining cells were stained and analyzed via flow cytometry. Graphed are the number of activated tumor specific TNF-α^+^IFN-γ^+^ CD8^+^ (**A**) and CD4^+^ (**B**) T cells in the blood (n = 6 mice per group). (**C**) Tumor specific antibodies in the serum from treated mice were quantified by flow cytometry and the mean fluorescent intensity (FITC channel) graphed (n = 6 mice per group). AOaV-1, avian orthoavulavirus-1; AAV, Adeno-associated virus; PBS, phosphate-buffered saline mock control group; 3TSR, thrombospondin-1 type I repeats; * *p* < 0.05; ** *p* < 0.01; ns = not significant. Error bars denote standard deviation.

Flow cytometry was conducted on blood samples that were harvested 10 days post AOaV-1 administration, around the peak of the T cell response, to quantify tumor specific T cells in the blood. The AAV-Bevacizumab+AOaV-1 treatment group had significantly more activated tumor specific CD8^+^ T cells in the blood compared to all other treatment groups that also received AOaV-1 (Figure 3A). In contrast to CD8^+^ T cells, there was no statistically significant difference between the number of CD4^+^ TNF-α^+^IFN-γ^+^ T cells (*p* = 0.1026–0.9968) (Figure 3B). There were no significant differences between the number of activated tumor specific CD4^+^ T cells or CD8^+^ T cells within the AAV monotherapy groups.

### 3.4. AAV-3TSR Monotherapy Resulted in Significantly Higher Tumor-Specific Antibodies

To quantify the number of tumor-specific antibodies in the serum of treated mice, blood samples were taken 30 days post AOaV-1 administration and analyzed using a flow cytometry assay developed in our lab [66] (Figure 3C). These data demonstrate that AAV-3TSR treatment yielded the highest concentration of tumor-specific antibodies in the serum, based on the mean fluorescent intensity of the FITC channel (MFI = 14,608), indicating more tumor specific B cell activation in the AAV-3TSR treatment group.

### 3.5. Analysis of Disease Progression at 90 Days Post Tumor Cell Implantation

**Figure 4:**
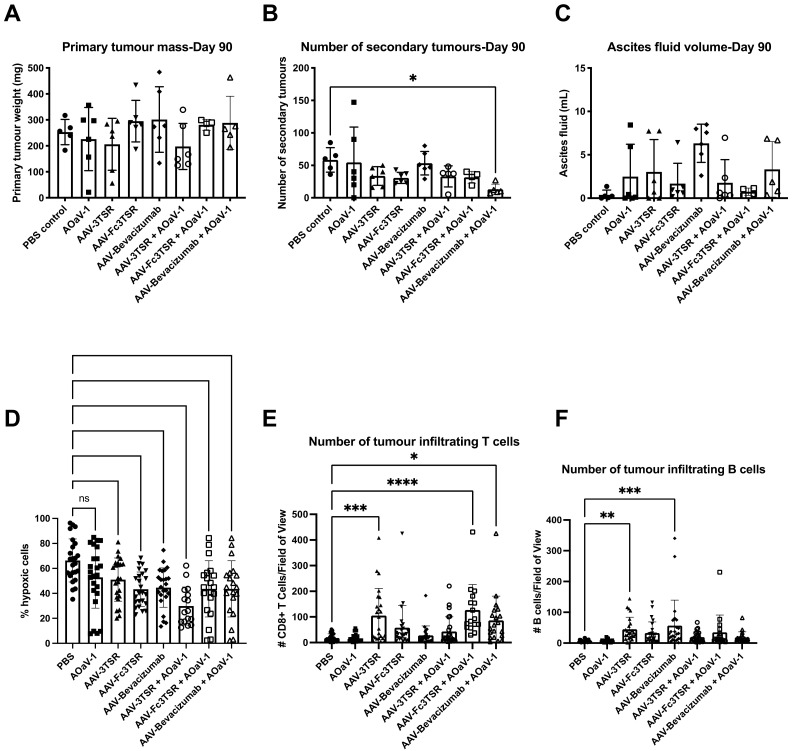
Analysis of disease progression at 90 days post ID8 tumor cell implantation. Groups of mice (n = 6) were analyzed 90 days post ID8 injection [50 days post i.m. administration of AAV-3TSR (1 × 10^11^ vg), Fc3TSR (1 × 10^11^ vg) and AAV-Bevacizumab (1 × 10^10^ vg) and 30 days post i.v. administration of 1 × 10^8^ PFU AOaV-1] to characterize the disease burden of the murine epithelial carcinoma model. (**A**) Primary ovarian tumors weight in grams. (**B**) The number of secondary tumors enumerated in the peritoneal cavity. Metastases were observed on the peritoneal cavity wall, diaphragm, liver, kidney, spleen, stomach, intestine, and pancreas. (**C**) Ascites was aspirated from the cavity of each mouse using a 22-gauge needle and the fluid volume recorded. (**D**) Analysis of primary tumor hypoxia. Vascular normalization was analyzed using an Hydroxyprobe kit. Mice were administered hydroxyprobe intraperitoneally 2 h prior to being euthanized. (**E**) Immunohistochemical analysis of intratumoral CD8^+^ T cells and (**F**) B cells. Primary tumors and tumor-draining lymph nodes were preserved in 10% formalin and paraffin embedded. CD4^+^ and CD8^+^ T cell infiltration were also analyzed. With AOaV-1, avian orthoavulavirus-1; AAV, Adeno-associated virus; PBS, phosphate-buffered saline mock control group; 3TSR, thrombospondin-1 type I repeats; * *p* < 0.05; ** *p* < 0.01; *** *p* < 0.001; **** *p* < 0.0001; ns = not significant. Error bars denote standard deviation. Closed circle, PBS; closed square, AOaV-1; closed triangle, AAV-3TSR; upside down triangle, AAV-Fc3TSR; diamond, AAV-Bevacizumab; open circle, AAV-3TSR+AOaV-1; open square, AAV-Fc3TSR+AOaV-1; open triangle, AAV-Bevacizumab+AOaV-1.

A subset of mice (n = 6) from each treatment group was euthanized 90 days post tumor-cell implantation in order to compare disease progression amongst the treatment groups prior to reaching endpoint. At 90 days post-tumor cell implantation, mice are typically moribund and have developed large primary tumors as well as secondary tumors within the peritoneal cavity, and extensive ascites fluid production [69]. The primary tumor weight (Figure 4A), number of secondary lesions (Figure 4B), and volume of ascites (Figure 4C) was quantified. Due to a large variation in the standard deviation, there were no significant differences in the primary tumor weights between experimental groups. However, mice treated with AAV-3TSR+AOaV-1 had primary tumors that weighed on average 22.1% less than PBS controls, while tumors in the AAV-3TSR and AOaV-1 monotherapy groups weighed on average 19.7% and 11.8% less than the controls, respectively. Although these reductions were not statistically significant, it is possible a one fifth reduction in tumor size by day 90 could contribute to extensions to survival. The PBS control group had 4.5× more metastases than the AAV-Bevacizumab+AOaV-1 treatment (*p* = 0.0300). There were no significant differences in ascites volume between the various treatment groups (Figure 4C).

The primary tumors and tumor draining lymph nodes were fixed, paraffin embedded, sectioned, and subjected to histological analysis. A Hydroxyprobe kit was used to measure hypoxia, while immunofluorescence staining was employed to quantify the number of tumor infiltrating CD8^+^ T and B cells. Hypoxia analysis confirmed that the AAV gene therapies were functioning to reduce hypoxia as expected. The PBS control had the greatest percentage of hypoxic cells (53.7 ± 17.2%) (Figure 4D). Staining for CD8^+^ T cells demonstrated that the PBS and AOaV-1 controls had the lowest numbers of CD8^+^ T cells in tumor sections (18.1 and 16.5 per field of view, respectively; Figure 4E). The AAV-3TSR monotherapy (105.2, *p* = 0.0003), AAV-Fc3TSR+AOaV-1 (126.9, *p* < 0.0001), and AAV-Bevacizumab+AOaV-1 (86.4, *p* = 0.0110) treatments all had significantly higher numbers of CD8^+^ T cells than the PBS control. Likewise, the PBS and AOaV-1 controls also had the lowest numbers of infiltrating B cells in tumor sections (6.2 each) (Figure 4F). The monotherapy treatments AAV-3TSR (45.1, *p* = 0.0700), and AAV-Bevacizumab (56.5, *p* = 0.0002) had significantly higher numbers of infiltrating B cells per field of view.

### 3.6. Serum Expression Levels of 3TSR, Fc3TSR and Bevacizumab Diminished More Rapidly in Tumor Bearing Mice Than in Naïve Mice

**Figure 5:**
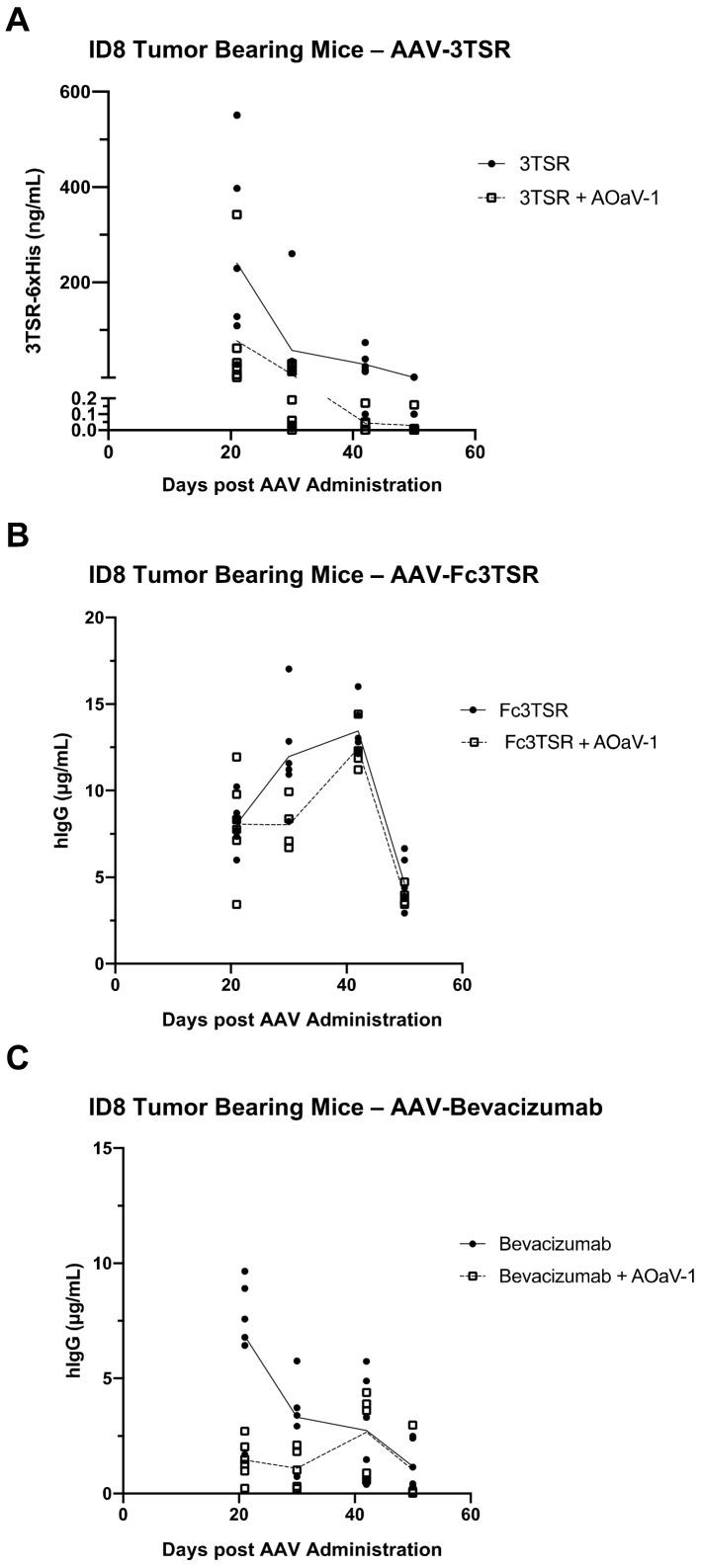
Serum expression levels of AAV-vectorized 3TSR, Fc3TSR, and Bevacizumab in ID8 tumor bearing mice. Serum samples from ID8 tumor bearing mice i.m. administered AAV-3TSR (1 × 10^11^ vg), Fc3TSR (1 × 10^11^ vg) or AAV-Bevacizumab (1 × 10^10^ vg) on day 40 post-tumor cell implantation and i.v. administered 1 × 10^8^ PFU AOaV-1 20 days later were collected weekly until the day 90 endpoint. AAV-expressed (**A**) 3TSR, (**B**) Fc3TSR, and (**C**) Bevacizumab was quantified in serum samples from ID8 tumor bearing mice using commercial ELISAs. With AOaV-1, avian orthoavulavirus-1; AAV, Adeno-associated virus; PBS, phosphate-buffered saline mock control group; 3TSR, thrombospondin-1 type I repeats; i.m., intramuscular injection; i.p., intraperitoneal cavity injection. Closed circle indicates AAV monotherapy. Open square indicates combination therapy of AAV plus AOaV-1.

The AAV-mediated expression of therapeutic transgenes in the serum of ID8 tumor bearing mice was measured on days 21, 30, 42, and 50 post AAV-administration (Figure 5). Unexpectedly, there was a more rapid decline in transgene expression compared to earlier expression kinetics of the same AAV vectors in naïve mice (Figure 1). Furthermore, AAV-Fc3TSR exhibited the highest serum Fc3TSR concentration on day 42 (13.44 ± 1.48 µg/mL), followed by a rapid decline by day 50 (4.62 ± 1.42 µg/mL). In the combination therapy AAV-Fc3TSR+AOaV-1 treatment group average serum Fc3TSR concentrations peak on day 42 (12.45 ± 1.38 µg/mL), and rapidly declined by day 50 (3.92 ± 0.59 µg/mL). AAV-3TSR and AAV-Bevacizumab had expression levels that decreased from day 21 onward (Figure 5A,C). Unexpectedly, and in contrast to AAV-Fc3TSR, the AAV-Bevacizumab+AOaV-1 combination therapy treatment group had significantly lower expression at day 21 compared to the monotherapy group (*p* < 0.0001), despite AAV doses being administered at the same time from the same vial. There was no significant difference between the two therapies as time progressed and the monotherapy lost expression over time (*p* = 0.2130). Additionally, AAV-3TSR monotherapy had a persistent decline in expression, being significantly lower at day 30 (*p* = 0.0091), day 42 (*p* = 0.0018), and day 50 (*p* = 0.0004) compared to day 21. There was a significant difference between the AAV-3TSR monotherapy and combination therapy only at day 21 (*p* = 0.0170), which dissipated as time progressed and AAV-3TSR expression decreased (*p* = 0.8612).

### 3.7. All Three AAV Treatments Significantly Extended Survival in the ID8 Ovarian Cancer Model with AAV-Bevacizumab Treatment Resulting in the Greatest Extension in Survival

A survival study was conducted (n = 8 mice per treatment group) to determine which of the three AAV gene therapy treatments resulted in the longest extension of survival in the ID8 orthotopic ovarian cancer model. The three monotherapy treatment options evaluated were AAV-3TSR, AAV-Fc3TSR, and AAV-Bevacizumab and the three combination therapies involved combining AAV therapies with oncolytic AOaV-1, which was administered 20 days after the gene therapies to ensure sufficient time for the transgenes to mediate their antiangiogenic functions. Additionally, PBS and AOaV-1 were included as controls.

**Figure 6:**
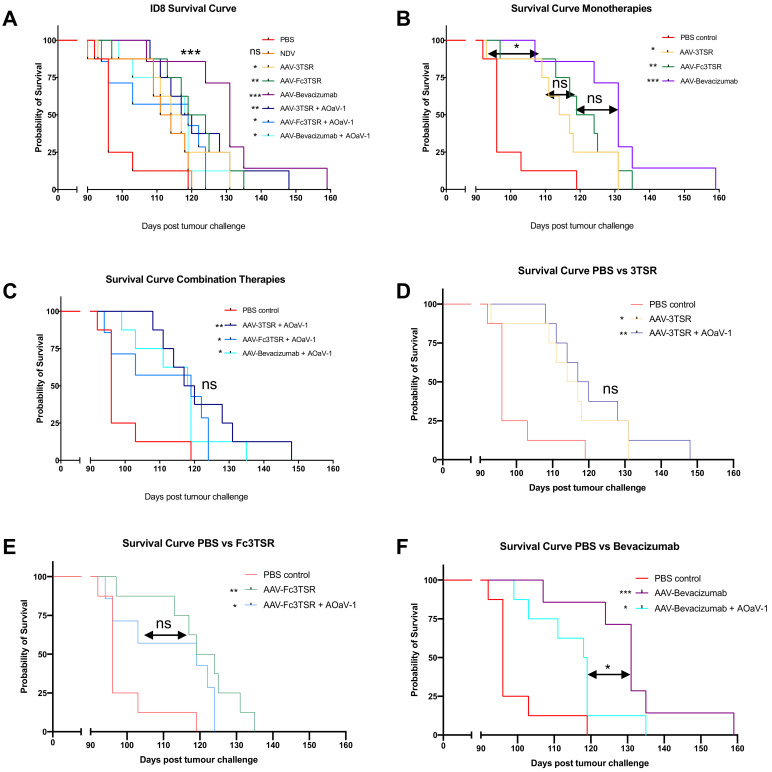
AAV vectorized expression of 3TSR, Fc3TSR and Bevacizumab increases survival in the ID8 mouse model of advanced stage epithelial ovarian carcinoma. (**A**) 64 mice were injected with 1 × 10^6^ ID8 ovarian epithelial carcinoma cells into the ovarian bursa. Forty days later, groups of mice (n = 8) were i.m. administered AAV-3TSR (1 × 10^11^ vg), Fc3TSR (1 × 10^11^ vg) or AAV-Bevacizumab (1 × 10^10^ vg) followed 20 days later by i.v administration of 1 × 10^8^ PFU AOaV-1. Mice were monitored daily for signs of ruffled fur, hunching, reduction in movement, and body score. Ascites was drained ~1 time per week as needed. The survival curve from panel A was separated into subcategories to facilitate comparisons of the gene therapies alone and in combination with AOaV-1. Statistics adjacent to the figure legends denote statistical significance between the specific treatment and PBS. (**B**) Monotherapies. (**C**) Combination therapies. (**D**) AAV-3TSR. (**E**) Fc3TSR. (**F**) AAV-Bevacizumab. AOaV-1, avian orthoavulavirus-1; AAV, Adeno-associated virus; PBS, phosphate-buffered saline mock control group; 3TSR, thrombospondin-1 type I repeats; * *p* < 0.05; ** *p* < 0.01; *** *p* < 0.001; ns = not significant.

All three of the AAV monotherapies significantly extended survival in this model of late state ovarian cancer (Figure 6A). The median survival for the PBS group was 96 days, with the final mouse reaching endpoint on day 119. The median survival for the AOaV-1 control group was longer than PBS at 112.5 days; however, the final mouse reached endpoint only a day later than PBS, at day 120. The monotherapies AAV-3TSR, AAV-Fc3TSR, and AAV-Bevacizumab resulted in a median survival of 115.5, 121.5, and 131 days, respectively, with the final mouse reaching endpoint on days 131, 135, and 159 (Figure 6B). The combination therapies with AOaV-1 resulted in similar median survivals of 118.5, 119, and 118.5 days, respectively with the final mouse reaching endpoint at day 148, 124, and 135 (Figure 6C–F). A single dose of AOaV-1 did not significantly extend overall survival in this ovarian cancer model (*p* = 0.0822) (Figure 6A). Moreover, there was no significant difference in survival when AOaV-1 was combined with AAV-3TSR (*p* = 0.5094) (Figure 6D) or AAV-Fc3TSR (*p* = 0.1617) (Figure 6E). Interestingly, AAV-Bevacizumab alone performed significantly better than when combined with AOaV-1 (*p* = 0.0496) (Figure 6F). Thus, within the parameters of this specific experiment, the AAV-Bevacizumab monotherapy was the most efficacious treatment (*p* = 0.0003).

## 4. Discussion

This study aimed to analyze the therapeutic efficacy of AAV-mediated expression of two vascular normalizing agents and one anti-angiogenic compound in a murine model of advanced stage ovarian epithelial carcinoma. These AAV gene therapies were tested both as monotherapies and in combination with oncolytic AOaV-1. This survival study (Figure 6) was combined with gene expression data in both naïve and tumor-bearing mice (Figure 1 and Figure 5) as well, the immunological and pathological impact of these therapies on the blood, lymph nodes, and primary tumor was investigated (Figure 4). All three of the AAV therapies significantly extended survival in this stringent model of EOC, with AAV-Bevacizumab monotherapy resulting in the longest survival extension of up to two months of additional life.

Preliminary studies in naïve mice demonstrated that all three AAV vectors elicited detectable levels of transgene expression with AAV-Bevacizumab and AAV-Fc3TSR reaching 100 and 10 fold higher concentrations, respectively, in the serum than AAV-3TSR (Figure 1). In naïve mice, AAV-Fc3TSR and AAV-Bevacizumab treated mice had detectable Fc3TSR and Bevacizumab in the serum and peritoneal cavity at day 211 when the mice were euthanized, although the concentration had decreased from peak expression levels, which occurred between 42–56 days post-AAV administration. In contrast, AAV-3TSR expression levels peaked at day 56 and then rapidly declined below the limit of detection by day 142. Note that a different ELISA with lower sensitivity was used to quantify 3TSR; therefore, it is possible that 3TSR was present in the serum at ng/mL quantities. Possible reasons as to why 3TSR was present in the serum at lower concentrations than Fc3TSR and Bevacizumab, and was more rapidly cleared from circulation include the fact that 3TSR is considerably smaller in size and does not contain an Fc domain, which can bind to the neonatal Fc receptor (FcRn) and facilitate recycling and protection from degradation [30,70]. Unexpectedly, the duration of transgene expression was markedly reduced when AAV vectors were administered to ID8 tumor bearing mice (Figure 5). The rapid decline in transgene expression occurred despite the gene therapies being from the same AAV6.2FF production batch. This observation has widespread implications for gene therapeutics for cancer treatment. There are several possibilities that could be hypothesized to explain this observation. Tumors have a higher metabolism [71,72], and it is plausible that this resulted in a quicker turnover of the vascular normalizing/antiangiogenic agents. Another possibility is that the primary tumor acted as a sink for the gene therapeutics, resulting in their more rapid turnover or sequestration [73]. Biodistribution studies of antibodies against the VEGF receptor NRP1 have concluded that saturation of non-tumor sinks was required to improve tumor exposure to the compound of interest [74]. Additionally, prostate cancer research has shown the link between total tumor volume and tumor sink effects for radioligand therapy [75]. Researchers aiming to use gene therapy vectors to express therapeutic transgenes for cancer treatment should therefore closely monitor transgene expression, as a second dose may be warranted sooner than in non-tumor-bearing subjects. A report comparing AAV2-mediated transgene expression levels in the brain, heart, kidney, liver, lung, muscle, and spleen of naïve and HeyA8 ovarian cancer bearing mice [76] noted several variations in expression levels between naïve and tumor bearing mice. Thus, it should be taken into consideration that tumor burden may impact levels of therapeutic transgene expression compared to studies conducted in naïve mice.

Our flow cytometry and IHC results demonstrate that the immune system was highly responsive to the various therapies analyzed (Figure 3 and Figure 4). We found that AAV-3TSR treated mice had the highest level of tumor specific antibodies in the blood, paired with increased B cells and CD8^+^ T cells in the tumor microenvironment. The AAV-Fc3TSR therapy resulted in the largest number of IFN-γ^+^ NK cells in the blood, but had decreased levels of tumor specific Abs compared to 3TSR. AAV-Bevacizumab treated mice displayed the highest number of TNF-α^+^IFN-γ^+^ tumor specific T cells in the blood, and increases in tumor infiltrating CD8^+^ T cells and B cells. AOaV-1 treatment had a significant impact on NK cells, dramatically increasing activation levels and creating an effector phenotype. The decrease in the number of Granzyme B^+^ NK cells in the blood of all mice receiving gene therapies may potentially be attributed to the cells trafficking to other anatomical regions, including the tumor after vascular normalization had occurred. Future experiments focused on quantifying immune cell activation within the tumor microenvironment and depleting NK and T cell populations would aid in understanding the mechanism by which these AAV-gene therapies contribute to efficacy in this model.

An unexpected result of this study was the conclusion that with this specific dosing schedule, administering AOaV-1 in combination with the AAV therapies did not improve survival (Figure 6). Based on the results of a previous study which demonstrated that administering AOaV-1 to ID8 tumor bearing mice receiving daily 3TSR treatments had a greater reduction in primary tumor mass, ascites accumulation, and secondary lesions than either treatment on its own [65], we would have anticipated a survival benefit in this study. The AOaV-1 that was administered was confirmed to be bioactive based on flow cytometry data obtained 36 h post AOaV-1 injection (Figure 2). As has been observed previously [67], AOaV-1-induced leukopenia characterized by transient a decrease in immune cell subsets in the blood. Within the NK cell population, numerous markers were upregulated for all combination therapies, notably CD69, PD-L1, NKG2D, NKp46, Granzyme B, and IFN-γ. NK cells had an activated phenotype upon AOaV-1 administration including upregulation of NKG2D, which can increase NK cell cytotoxicity [77], and upregulation of NKp46, which is involved in recognition of both tumor cells and viruses [78]. AAV-Bevacizumab in combination with AOaV-1 led to a significant increase in the number of tumor-specific TNF-α^+^IFN-γ^+^CD8^+^ T cells, which have previously been shown to be a beneficial subset in cancer immunotherapies. Although AOaV-1 did not mediate a survival benefit when used in combination with vectorized antiangiogenic agents, the positive impact on NK cells and tumor specific T cells upon administration suggests that additional doses of AOaV-1 may be required to increase the therapeutic efficacy of this combination therapy. Experiments are currently underway to address this hypothesis.

One possible explanation for the lack of combination therapy survival extension, accompanying IHC and day 90 necropsy results may be attributed to large differences in serum concentrations of the vascular normalizing agents between the gene therapies versus when the drugs are administered daily or weekly as recombinant proteins. Noted differences in the serum concentrations of the vascular normalizing agents exists between administration of recombinant proteins (Figure S1) and their AAV-vectorized versions. For example, when Fc3TSR was administered to mice as a recombinant protein, the serum concentrations were much lower (ng/mL concentrations compared to µg/mL quantities), and decreased significantly within seven days post injection. A major reason for testing the gene therapy versions of each compound was to avoid the need for daily or weekly injections, as a single dose mediating long-term sustained expression would be a preferable option for patients. It is plausible that administration of AAV-3TSR and AAV-Fc3TSR at 1 × 10^11^ vg/mouse i.m. and Bevacizumab at 1 × 10^10^ vg/mouse i.m. (lower dose to mimic the same magnitude of transgene expression as Fc3TSR) led to expression levels that were higher than the optimal dose for tumor reduction, thus exceeding the narrow U-shaped optimal therapeutic window of TSP-1 and endostatins [26,79,80,81]. Perhaps in the case of this ovarian cancer model, less is more. It is possible that either administering the same gene therapies i.p. at these concentrations or administering i.m. at a half or 1/10th the dose may have resulted in further extensions to survival and reductions in tumor size and secondary metastases at day 90. Testing the effects of i.p. administration in combination with AOaV-1, as well as lower doses of i.m. in combination with AOaV-1, constitutes a very promising area of future research.

In conclusion, this study set out to test three different gene therapies expressing antiangiogenic agents from AAV to treat advanced stage ovarian epithelial carcinoma, using an orthotopic preclinical mouse model. All three AAV gene therapies led to a significant extension in survival, including combination therapies that use the oncolytic virus AOaV-1. Indeed, the monotherapy AAV-Bevacizumab resulted in mice living up to two months longer than in the PBS controls. Although the combination therapies did not result in a significant improvement upon the monotherapies, our data suggest that future studies combining lower doses of the gene therapeutics with additional doses of AOaV-1 may result in further improvements to therapeutic efficacy. 

In summary, our data demonstrate the potential use of AAV vectors in the application of anti-angiogenic strategies to treat ovarian cancer, potentially in combination with other therapeutic interventions such as oncolytic viruses. Additional studies are warranted to further explore the AAV-vectorized expression of antiangiogenic compounds in different cancer models and, eventually, in human clinical trials.

## Data Availability

All data is contained within the article and Supplementary Materials.

## Supplementary Materials

The following are available online at https://www.mdpi.com/article/10.3390/biomedicines10020362/s1, Figure S1: Serum levels of Fc3TSR, Figure S2: Flow cytometry gating strategy.
