# Supplementary material for: AAV-Vectored Expression of the Vascular Normalizing Agents 3TSR and Fc3TSR, and the Anti-Angiogenic Bevacizumab Extends Survival in a Murine Model of End-Stage Epithelial Ovarian Carcinoma
Source: Biomedicines. 2022 Feb 2;10(2):362. doi: 10.3390/biomedicines10020362 (PMC8962366; doi:10.3390/biomedicines10020362)
Supplement: Supplementary file 1 [file biomedicines-10-00362-s001.zip › biomedicines-1519023-supplementary.pdf]

biomedicines

MDPI

Supplementary Material

AAV-vectored expression of the vascular normalizing agents 3TSR and Fc3TSR, and the anti-angiogenic Bevacizumab extends survival in a murine model of end-stage epithelial ovarian carcinoma

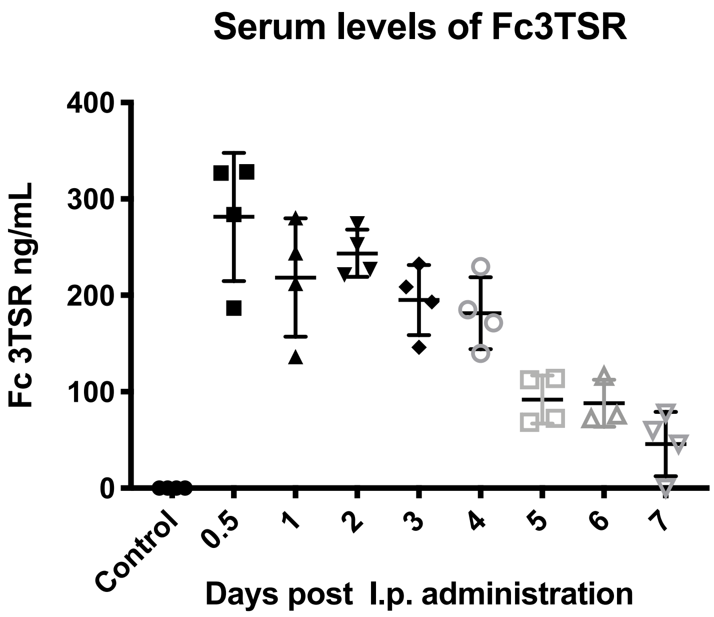

Figure S1. Serum levels of Fc3TSR. The recombinant protein version of the drug Fc3TSR was injected via intraperitoneal administration. Serum levels were monitored over 7 days to quantify the gradual decline in serum concentrations. i.p., intraperitoneal administration route; 3TSR, thrombospondin-1 type I repeats. Error bars denote standard deviation.

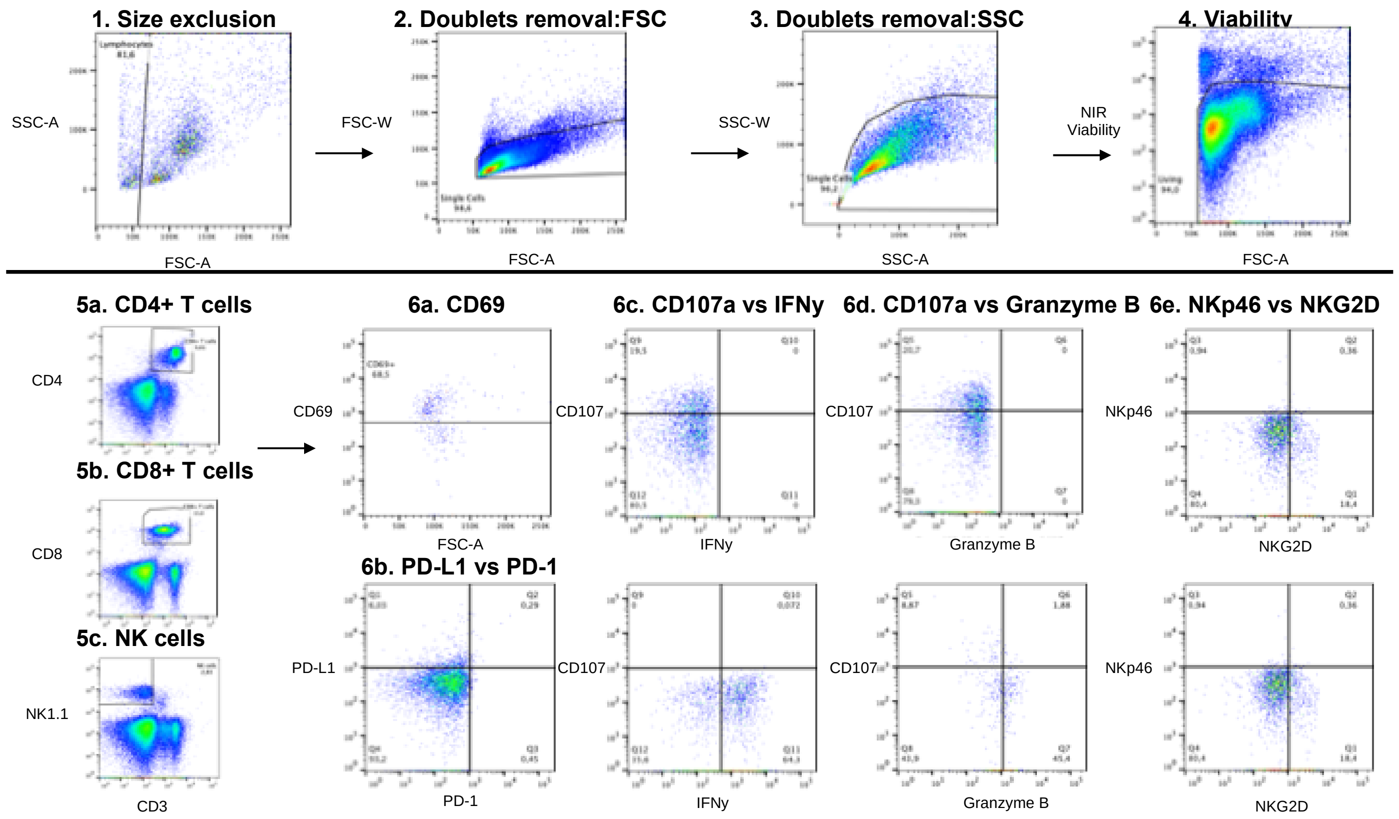

Figure S2. Flow cytometry gating strategy. Data was analyzed using FlowJo software. Cells were initially gated based on size. Subsequently, doublets were removed as well as non-viable cells based on Zombie NIR Fixable Viability Dye Staining. $CD4^+$ T cells were defined as $CD3^+$ $CD4^+$. $CD8^+$ T cells were defined as $CD3^+CD8^+$. NK cells were defined as CD3-$NK1.1^+$. Each of these three immune cell subsets were analyzed for expression levels of the early activation marker CD69, and the immune checkpoint proteins programmed death ligand 1 (PD-L1) and programmed cell death protein 1 (PD-1). NK cells were further analyzed for expression levels of the serine protease Granzyme B, the anti-viral IFN-γ, the NK functional marker CD107a, the natural cytotoxicity receptor NKp46, and the cytotoxic activating receptor NKG2D.
